# Discovery of targetable genetic alterations in advanced non-small cell lung cancer using a next-generation sequencing-based circulating tumor DNA assay

**DOI:** 10.1038/s41598-017-14962-0

**Published:** 2017-11-03

**Authors:** Helei Hou, Xiaonan Yang, Jinping Zhang, Zhe Zhang, Xiaomei Xu, Xiaoping Zhang, Chuantao Zhang, Dong Liu, Weihua Yan, Na Zhou, Hongmei Zhu, Zhaoyang Qian, Zhuokun Li, Xiaochun Zhang

**Affiliations:** 1 Department of Medical Oncology, The Affiliated Hospital of Qingdao University, Qingdao University, 16 Jiangsu Road, Qingdao, 266005 China; 2 BGI-Qingdao Institute, Qingdao SINO-GERMAN Ecopark, 2877 Tuanjie Road, Qingdao, 266555 China; 3 Department of Experimental Therapeutics, University of Texas, South Campus Research Building 4 (4SCR), Room 4SCR3.2085, 1901 East Road, Houston, Texas 77054 USA; 4 Department of Thoracic Surgery, Qingdao Municipal Hospital, 1 Jiaozhou Road, Qingdao, 266011 China; 5 Department of Medical Oncology, Qingdao Municipal Hospital, 5 Donghai Middle Road, Qingdao, 266071 China; 6 BGI-Shenzhen, Shenzhen, 518083 China; 7 Department of Pathology, The Affiliated Hospital of Qingdao University, Qingdao University, 16 Jiangsu Road, Qingdao, 266005 China; 8 Binhai Genomics Institute, BGI-Tianjin, BGI-Shenzhen, Tianjin, 300308 China

## Abstract

Next-generation sequencing (NGS)-based circulating tumor DNA (ctDNA) assays have provided a new method of identifying tumor-driving genes in patients with advanced non-small cell lung carcinoma (NSCLC), especially in those whose cancer tissues are unavailable or in those that have acquired treatment resistance. Here, we describe a total of 119 patients with advanced EGFR-TKI-naive NSCLC and 15 EGFR-TKI-resistant patients to identify somatic SNVs, small indels, CNVs and gene fusions in 508 tumor-related genes. Somatic ctDNA mutations were detected in 82.8% (111/134) of patients in the total cohort. Of the 119 patients with advanced NSCLC, 27.7% (33/119) were suitable for treatment with National Comprehensive Cancer Network (NCCN) guideline-approved targeted drugs. Actionable genetic alterations included 25 EGFR mutations, 5 BRAF mutations, and 1 MET mutation, as well as 1 EML4-ALK gene fusion and 1 KIF5B-RET gene fusion. In 19.3% (23/119) of the patients, we also identified genomic alterations with that could be targeted by agents that are in clinical trials, such as mTOR inhibitors, PARP inhibitors, and CDK4/6 inhibitors. Additionally, the EGFR T790M mutation was found in 46.7% (7/15) of the patients with EGFR-TKI-resistant NSCLC, suggesting that the NGS-based ctDNA assay might be an optional method to monitor EGFR-TKI resistance and to discover mechanisms of drug resistance.

## Introduction

Most tumors are discovered to be locally advanced or metastatic, as is the case for lung cancer, which is a prevailing cause of death worldwide^1^. With advances in molecular diagnosis and targeted therapies, molecular genotyping is now routinely used to guide the clinical treatment of patients with non-small cell lung carcinoma (NSCLC). The efficacy of targeted kinase inhibitors was demonstrated to be superior to that of standard chemotherapy for patients with EGFR mutations or ALK/Ros1 fusions^2^. In addition, NSCLC frequently harbors genomic alterations in KRAS, BRAF, ERBB2, RET and MET. Potential targeted agents for these genomic mutations are available from an ongoing trial or are being used off protocol^2^.

Currently, ARMS PCR, Sanger sequencing and FISH are commonly used to detect a few targetable oncogenes and hotspot mutations^3^. However, such assays are insufficient since most of these genes are not altered in a large proportion of patients. In terms of the complex genomic alterations in NSCLC, there is an urgent need to screen potentially actionable targets simultaneously. Next-generation sequencing (NGS) has revolutionized molecular diagnostics, and enabled the simultaneous detection of multiple alterations in a single test. NGS-based hybrid capture assays not only allow the identification of hotspot mutations but also allow the assessment of unknown alterations, all from a single formalin-fixed, paraffin-embedded (FFPE) specimen or serum sample^4^.

Circulating tumor DNAs (ctDNAs), which carry tumor-specific sequence alterations, represent a variable and generally small fraction of the total circulating DNA^5^. Studies have shown that ctDNA is an informative, inherently specific and highly sensitive biomarker of metastatic breast cancer^6^. Analysis of ctDNA is particularly attractive for those patients without enough tissue samples or those who cannot be repeatedly sampled with invasive procedures after disease progression. In NSCLC, both non-NGS-based and NGS-based assays with variable sensitivity have been used to detect genomic alterations in serum samples^7,8^. The detection of ctDNA in NSCLC could be used to guide targeted therapy, identify resistance mechanisms, and monitor clinical prognosis^9–11^. Most of the ctDNA detection approaches are limited to hotspot mutations in a few genes. By comparison, NGS-based ctDNA assays offer the ability to profile a much broader range of genetic alterations in a single test. To date, studies have tried to apply ctDNA NGS panels that screen from 70 to 252 genes for the detection of clinically actionable variants and resistance mutations in patients with lung cancer^12,13^.

In this study, a broad hybrid capture-based 508-gene panel NGS assay (Oseq-NT) was used to screen targetable genomic alterations of ctDNA from patients with NSCLC. We intended to confirm the potential benefits of this ctDNA detection method in guiding personalized therapy in patients with NSCLC.

## Materials and Methods

### Patients and Samples

We analyzed 119 patients with stage IIB-IV NSCLC and 15 patients who had developed drug-resistance to EGFR-TKIs. Patient characteristics were shown in Table 1. The diagnosis was verified by fine-needle aspirations or cell pathology of pleural effusion before any therapy. Peripheral blood sample collections (10 ml) were approved by the Ethics Committee of the Affiliated Hospital of Qingdao University, and all patients signed informed consent. All the experiments were carried out in accordance with the guideline released by the National Health and Family Planning Commission of the PRC.

**Table 1:** Clinical characteristics of the 119 NSCLC patients.

Clinical characteristics (n = 119)	
Age-years	
Median	59.7
Range	31–94
Sex-no.	
Male	64(53.8%)
Female	55(46.2%)
Cigarette smoking status-no.	
Never smoked	51(42.9%)
Former smoker	68(57.1%)
Histologic type-no.	
Adenocarcinoma	105(88.2%)
Squamous cell carcinoma	14(11.8%)
Stage	
IIIB	6(5.0%)
IV	113(95.0%)

### NGS-based ctDNA assay

Circulating DNA was isolated from 2 mL plasma with the QIAamp Circulating Nucleic Acid Kit (Qiagen) according to the manufacturer’s instructions. Genomic DNA from peripheral blood was purified using QIAamp DNA Blood Mini Kit (Qiagen). DNA purity and concentration were examined by the NanoDrop2000 spectrophotometer and Qubit 2.0 Fluorometer with Quant-IT dsDNA HS Assay Kit (Thermo Fisher Scientific), respectively. The quality of genomic DNA from peripheral blood was assessed by agarose gel electrophoresis and the size distribution of circulating DNA was evaluated on a 2100 Bioanalyzer using the DNA 1000 Kit (Agilent).

Library construction with peripheral blood DNA was performed as previously described using 1mg of DNA sheared by an ultrasonoscope to generate fragments with a peak of 250 bps, followed by end repair, A-tailing and ligation to the Illumina-indexed adapters according to the standard library construction protocol. Target enrichment was performed on a custom sequence capture-probe (Nimblegen, USA) which targeted 7,708 exons of 508 cancer-related genes and 78 introns from 19 genes recurrently rearranged in solid tumor representing totaling ~1.7Mb of the human genome (Supplement Table S1). Library for circulating DNA was constructed by KAPA LTP Library Preparation Kit for Illumina Platform (Kapa Biosystems) following the manufacturer’s instructions without modification. Sequencing was performed with 2 × 101 bp paired-end reads and 8-bp index read on an Illumina Hiseq. 2500 plateform (Illumina, San Diego, USA).

Raw reads were first processed by removing adaptors and filtering low-quality ones using SOAPnuke (http://soap.genomics.org.cn/). Clean reads were then aligned to the human reference GRCh37 using BWA aligner (v0.6.2-r126)^14^. PCR duplication were removed by PICARD (v1.98). Local realignment and base quality score recalibration were performed using GATK (v2.3–9)^15^, based on which we removed poorly mapped reads. Then we identified SNVs using Mutect and SOMATK-SNV (developed by BGI, manuscript in preparation), and InDels were detected using GATK and SOMATK-INDEL (developed by BGI, manuscript in preparation). CNV calling was applied by CONTRA (v2.0.4)^16^. The CNV analysis was performed based on off-target sequencing data, which is used as low-depth whole genome sequencing data described in research by Bellos E *et al*.^17^. We split the whole genome into bins with 500kb length and count the read depth (RD) in each bin, followed by GC normalization method described by Yu Z *et al*.^18^. Bic-seq^19^ was used for segmentation of off-target sequencing data.

### Statistical analysis

The experimental data are presented as the mean ± SEM and were analyzed with the two-tailed Student’s *t* test. The threshold of *P* < 0.05 was considered as statistically significant.

## Results

### Use of the NGS-based ctDNA assay to screen 119 patients with advanced NSCLC

The clinical and pathological features of the patients are summarized in Table 1. The median age at diagnosis was 59.7 years with a range of 31–94, and 57.1% of the patients were smokers (68 of 119). Screening of the patients’ ctDNA identified somatic mutations in a total of 189 genes (with a mean of 5.5 ± 5.4 mutations per patient). Of all the samples, 81.5% (97/119) exhibited at least one genetic alteration and 18.5% (22/119) of samples exhibited no detectable alterations. The median average sequencing depth was 950 × for cell-free DNA from each sample and the basic summary of the genomic sequencing can be found in Supplementary Table S2.

**Figure 1:**
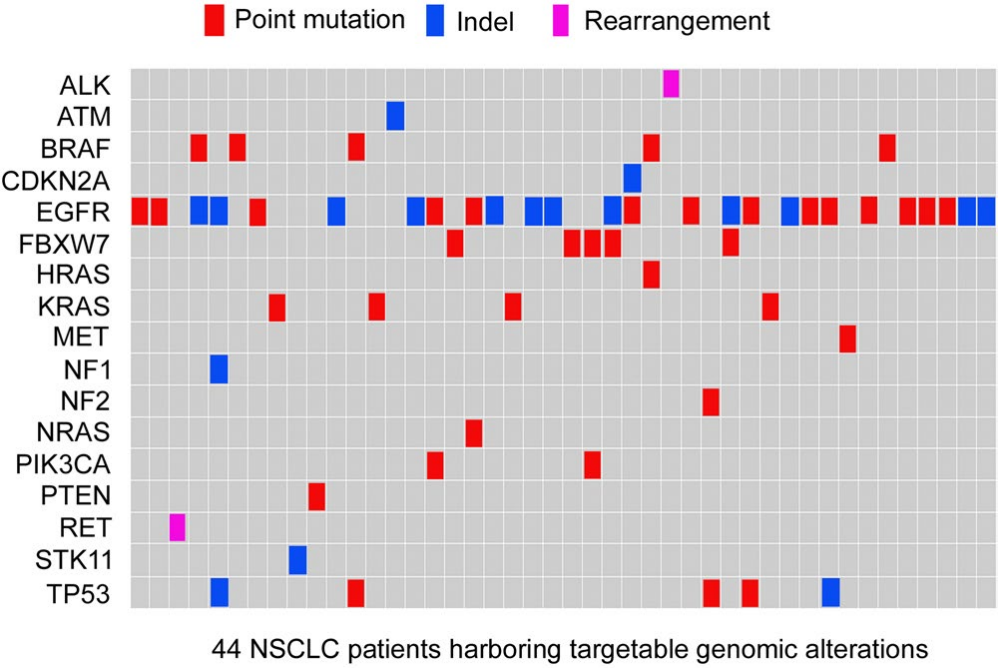
Comprehensive annotation of 44 NSCLC patients harboring targetable genomic alterations.

In total, 37.0% (44/119) of the patients with NSCLC had at least one targetable alteration (mean 1.38 ± 0.53) (Fig. 1), and the frequency of each specific alteration ranged from 0.5% to 56.9% (Supplementary Table S3). In terms of pathology type, targetable alterations were found in 37.1% (39/105) of the patients with adenocarcinoma and in 35.7% (5/14) of the patients with squamous cell carcinoma. Genomic alterations with corresponding targeted agents approved by the NCCN guidelines for NSCLC were identified in 27.7% (33/119) of the patients (Table 2). These actionable alterations included EGFR (n = 25), BRAF (n = 5), EML4-ALK (n = 1), KIF5B-RET (n = 1), and MET (n = 1). In addition, actionable genomic alterations corresponded to targeted therapy options that were available from ongoing trials or are being used off protocol were identified in another 19.3% (23/119) of the patients (Table 2). The most common alterations identified in our analysis included those in K-RAS (n = 4), TP53 (n = 5), N-ras (n = 1), ATM (n = 1) and CDKN2A (n = 1). Notably, we also identified 6 mutated genes that encode proteins involved in the mTOR pathway: namely, FBXW7 (n = 5), PIK3CA (n = 2), PTEN (n = 1), NF1 (n = 1), NF2 (n = 1) and STK11 (n = 1).

**Table 2:** Targetable ctDNA alterations found in 44 of the 119 NSCLC patients by NGS-based assay.

Gene	Alterations	n	Targeted agents
EGFR	p.G719A	2	EGFR-TKIs
EGFR	p.I740_K745dup, p.E746_A750del, p.E746_T751delinsI	8	EGFR-TKIs
EGFR	p.S768I	1	EGFR-TKIs
EGFR	p.L858R	5	EGFR-TKIs
EGFR	p.S768I + p.H835L	1	EGFR-TKIs
EGFR	p.L833V + p.L858R	1	EGFR-TKIs
EGFR	p.A767_V769dup	1	Resistant to EGFR-TKIs
FBXW7	p.R505H		mTOR inhibitors
EGFR	p.E746_P753delinsVS	1	Resistant to EGFR-TKIs
BRAF	p.V600E		Vemurafenib
EGFR	p.E746_A750del	1	EGFR-TKIs
FBXW7	p.R505S		mTOR inhibitors
EGFR	p.E746_A750del	1	EGFR-TKIs
NF1	p.S1030Ifs*8		mTOR inhibitors
TP53	p.P80Gfs*65		APR-246, MK-1775
EGFR	p.L858R	1	Resistant to EGFR-TKIs
NRAS	p.Q61K		MEK inhibitors
EGFR	p.L858R	1	EGFR-TKIs
PIK3CA	p.H1047R		mTOR inhibitors
EGFR	p.L858R	1	EGFR-TKIs
TP53	p.R342*		APR-246, MK-1775
ALK	EML4-ALK	1	Crizotinib
MET	p.Y1248H	1	Crizotinib
RET	KIF5B-RET	1	Cabozantinib
BRAF	p.V600E, p.G469V	2	Vemurafenib
BRAF	p.G469A	1	Vemurafenib
TP53	p.C141*		APR-246, MK-1775
BRAF	p.N581S	1	Vemurafenib
ATM	p.E2449*	1	PARP inhibitors
FBXW7	p.R505H, p.R505H + p.R465H	2	mTOR inhibitors
FBXW7	p.R505H	1	mTOR inhibitors
PIK3CA	p.M1043I		
KRAS	p.G12C, p.G12D	4	MEK inhibitors
NF2	p.Q111*	1	mTOR inhibitors
TP53	p.S183*		APR-246, MK-1775
PTEN	p.R130*	1	mTOR inhibitors
STK11	p.Y60Lfs*103	1	mTOR inhibitors
TP53	p.N239Ifs*7	1	APR-246, MK-1775

Numerous other alterations with no available targeted drugs were also observed in our cohort. Mutations in the following ten genes were observed in more than 5 samples: TP53, EGFR, FBXW7, BRAF, MLL2, MLL3, NAV3, K-RAS, FAT3 and TRRAP. Of these, no targeted agents have been reported for MLL2, MLL3, NAV3, FAT3, and TRRAP. The most frequent mutant genes were TP53 (n = 49, 41.2%) and EGFR (n = 30, 25.2%).

### The EGFR mutation spectrum and concomitant actionable genetic alterations

**Figure 2:**
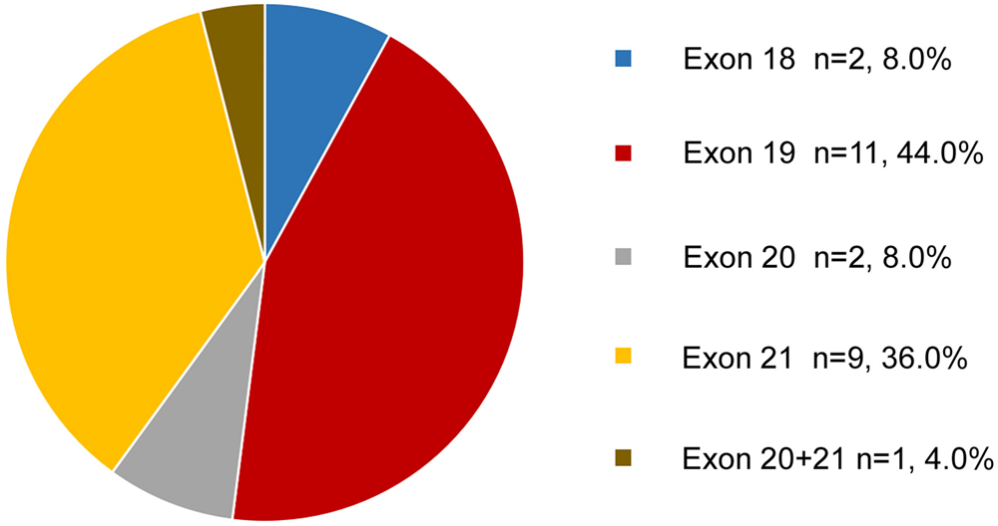
EGFR mutation spectrum in this 25 NSCLC patients.

Of the 119 patients with NSCLC, 25 (21.0%) were found to harbor actionable EGFR mutations. It should be noted that EGFR mutations were detected in 21.9% (23/105) of the patients with adenocarcinoma and 14.3% (2/14) of the patients with squamous cell carcinoma. The EGFR mutations in these 25 patients with NSCLC were located in the following exons: exon 18 (n = 2), exon 19 (n = 11), exon 20 (n = 2), exon 21 (n = 9), and exons 20 and 21 (n = 1) (Fig. 2). Notably, 7 of the 25 patients were found to harbor alternative actionable genetic alterations in addition to the EGFR mutation. Concomitant BRAF or NRAS mutations were found in two patients. In 4 patients, an EGFR mutation was accompanied with a mutation in an mTOR pathway-related gene (2FBXW7, 1NF1, and 1PTEN) (Table 2).

### An NGS-based ctDNA assay at the time of acquired EGFR-TKI resistance

Fifteen patients with NSCLC who had developed resistance to EGFR-TKIs underwent blood sampling and NGS-ctDNA assays **(**Table 3**)**. Tissue sample analysis confirmed that all these patients harbored EGFR-activating mutations before EGFR-TKI treatment. Of the 15 plasma samples obtained from patients post-EGFR-TKI treatment, EGFR-sensitive mutations were detected in 10 patients, which was consistent with prior tissue-based assays. Noticeably, the EGFR T790M mutation was also identified in 46.7% (7/15) of the patients. The allele frequency of the T790M mutation in this study varied from 0.8 to 28.3%. More importantly, the following mutations, which might be involved in EGFR-TKI resistance, were each identified in 1 patient: ERBB2 L755S, NRAS Q61K and EGFR amplification.

**Table 3:** NGS-based ctDNA assay at the time of acquired EGFR-TKIs resistance.

Patient	Prior EGFR tissue assay	EGFR-TKIs	Actionable mutations by NGS ctDNA assay after EGFR-TKIs resistance	mutation frequency
P02	p.E746_A750del /Sanger	Gefitinib	None	
P03	p.S768I/ARMS-PCR	Erlotinib	None	
P05	p.L858R/Sanger	Icotinib	EGFR p.L858R	2.2%
P14	p.L858R/ARMS-PCR	Gefitinib	EGFR p.L858R	2.29%
			EGFR p.T790M	0.94%
			NRAS p.Q61K	0.5%
P24	p.L858R/ARMS-PCR	Gefitinib	EGFR p.L858R	5.6%
			EGFR p.T790M	1.0%
P27	p.E746_A750del/ARMS-PCR	Gefitinib	EGFR p.E746_A750del	34.1%
			ERBB2 p.L755S	3.2%
P29	p.L858R/ARMS-PCR	Gefitinib	EGFR p.L858R	5.9%
			EGFR p.T790M	1.6%
P40	p.E746_A750del/ARMS-PCR	Gefitinib	EGFR p.E746_A750del	14.2%
			EGFR p.T790M	3.8%
P67	p.G719A/Sanger	Gefitinib	EGFR p.G719A	27.7%
			EGFR amplification	—
P68	p.E746_A750del/ARMS-PCR	Gefitinib	EGFR p.E746_A750del	28.5%
			EGFR p.T790M	28.3%
P74	p.L858R/ARMS-PCR	Gefitinib	EGFR p.L858R	2.4%
			EGFR p.T790M	2.7%
P75	p.E746_A750del/Sanger	Icotinib	None	
P77	p.E746_A750del/ARMS-PCR	Gefitinib	None	
P87	p.L858R/ARMS-PCR	Gefitinib	EGFR p.L858R	1.0%
			EGFR p.T790M	0.8%
P95	p.E746_A750del/Sanger	Icotinib	None	

## Discussion

The identification of specific molecular targets in NSCLC has led to the development of oncogene-directed targeted therapies. At present, several methods have been used to identify targetable mutations, and these methods have mainly analyzed cancer tissue samples^20,21^. However, it is often difficult to obtain sufficient tumor samples for genetic analysis in clinical practice. For patients who develop resistance to targeted therapy, molecular analysis is urgently required to identify the prevalence of both known and new resistance mutations that occur during therapy^22^. Under these circumstances, a noninvasive and real-time genomic detection assay is particularly valuable. In this study, we intended to discover actionable genomic alterations using an NGS-based ctDNA assay in patients with advanced NSCLC so that approved drugs or investigational agents in clinical trials could be selected. Through the study of 119 patients, ctDNA screening identified somatic mutations in a total of 189 genes across all patients, and somatic mutations were present in 81.5% of the patients. More importantly, 37.0% (44/119) of patients with NSCLC harbored at least one targetable alteration.

The detection and monitoring of tumors via noninvasive methods is a major challenge in oncology. ctDNA is composed of small fragments of nucleic acid that could be released into the circulation from CTCs and/or cancer tissues. Through the use of digital PCR technologies, Bettegowda *et al*. detected ctDNA in most patients with advanced cancers^23^. ctDNA might be a broadly applicable, sensitive, and specific biomarker that could be used for clinical purposes in patients with multiple different types of cancer^24,25^. In NSCLC, Newman *et al*. detected ctDNA in 100% of patients with stage II-IV NSCLC and in 50% of patients with stage I NSCLC using deep sequencing (CAPP-seq)^26^. In this study, somatic mutations were detected in the ctDNA of 97 of the 119 (81.5%) patients with advanced NSCLC using a broad, hybrid capture-based NGS assay (Oseq-NT) that has sufficient sensitivity and clinical applicability.

In advanced NSCLC, patients who have genomic alterations that are matched a targeted therapy have been found to live substantially longer than do those without such alterations^27^. However, largely due to limited tissue resources, not all the patients in this previous study could be analyzed by a genomic assay. Through the use of an NGS-based ctDNA assay in the present study, genomic alterations were identified in 27.7% of patients, and included alterations in EGFR, ALK, RET, BRAF, and MET. Consequently, these patients could receive corresponding targeted agents approved by the NCCN guidelines. In plasma DNA samples of Chinese patients with advanced adenocarcinoma, Qin *et al*. compared three methods for detecting EGFR mutations. EGFR mutations were detected in 6.9% samples by direct DNA sequencing, in 30.1% by denaturing high-performance liquid chromatography, and in 38.4% by Scorpions ARMS-PCR^28^. In contrast, EGFR mutations were detected in 21.9% (23/105) of adenocarcinoma samples by the NGS-based ctDNA assay in this study, which is a much higher proportion than detected by direct DNA sequencing yet a lower proportion than identified by Scorpions ARMS-PCR in the previous study. In addition, EGFR mutations were detected in 40.0% of non-paired tissue samples by the same NGS method (data not shown). Consequently, the approximate sensitivity of the NGS-based EGFR ctDNA assay reached 54.8%, and this needs to be improved by increasing the sequencing depth in the future.

Notably, our research discovered an additional 19.3% actionable genomic alterations for which targeted agents are available from ongoing clinical trials or are being used off protocol. The NCCN NSCLC Guidelines Panel strongly endorses broader molecular profiling with the goals of identifying rare driver mutations for which effective drugs may already be available and appropriately counselling patients regarding the availability of clinical trials. A study by Drilon *et al*. found that broad, hybrid capture-based NGS approaches could identify actionable genomic alterations in 65% of tumors from lung cancers that had been shown to lack targetable genomic alterations by earlier extensive non-NGS testing^4^. First-line profiling of lung adenocarcinomas using broad, hybrid capture-based NGS might be a more comprehensive and efficient strategy than is non-NGS testing. Therefore, NGS combined with a Scorpions ARMS-PCR ctDNA assay is a very promising diagnostic regimen for patients with NSCLC for whom limited tissue samples are available. Numerous other alterations with no available targeted drugs were also observed. The frequencies of mutations in several genes, including TP53, EGFR, FBXW7, BRAF and K-RAS, were consistent with previous massively parallel sequencing studies in lung adenocarcinoma^29^.

Although the application of TKI therapy to patients with EGFR-mutant NSCLC has led to a dramatic lengthening of both progression-free survival (PFS) and overall survival (OS), nearly all patients ultimately progress while receiving EGFR-TKI treatment. EGFR T790M has been identified as the most common mechanism of acquired resistance, and other, less frequent mechanisms have also been reported, including MET amplification, HER2 amplification, mutation of PIK3CA and BRAF, transformation to small-cell lung cancer and epithelial-to-mesenchymal transition^30^. The mechanism of EGFR-TKI resistance will determine the next treatment required, which emphasizes the need to repeat the biopsy and carry out molecular characterization of tumor samples at clinical progression. However, it is challenging to obtain serial tumor rebiopsies following disease progression in clinical practice owing to the invasiveness of the procedure and tumor heterogeneity. Several studies have assessed the ability of different technology platforms to detect EGFR mutations, including T790M^7,9,26^, in ctDNA. Detection rates of T790M ctDNA in plasma from patients post-EGFR-TKI treatment ranged from 43 to 47%, as determined by digital PCR^21,31^. In this study, EGFR T790M was detected in 46.7% of the patients with EGFR-TKI-resistant NSCLC by our NGS assay. Our results indicated that NGS was not inferior to digital PCR at detecting T790M in ctDNA. Interestingly, 3 EGFR-TKI resistance-related genetic alterations aside from EGFR-790M were identified in this study by the NGS assay: namely, ERBB2 L755S, NRAS Q61K and EGFR amplification. All of these EGFR-TKI-resistant genetic alterations might be missed by low-throughout non-NGS tests, which suggests the NGS ctDNA assay might be a more sensitive and comprehensive approach for drug resistance surveillance.

Recently, the most commonly used methods to detect EGFR mutations in ctDNA from patients with NSCLC depends on PCR-based techniques, and there has been a recent emergence of digital PCR and NGS. Representative studies are displayed in Table 4. Compared with these studies, the NGS-based ctDNA assay used in this study could identify more targeted alterations in addition to EGFR and could disclose EGFR-TKI resistance-related mutations aside from EGFR T790M. NGS-based ctDNA assays, especially with large gene panels, have a great advantage in guiding personalized treatment considering the growing number of therapeutic targets and known genetic alterations that confer resistance in NSCLC.

**Table 4:** Researches of ctDNA detection of EGFR mutation and other targeted alterations in NSCLC.

Patients No.	Material	Methods	Dectected genes	EGFR positivity rate in ctDNA	EGFR positivity rate in tissue DNA	Sensitivity	Specificity	Correpondence rate	Year/Ref
230	Plasma	DHPLC	EGFR exon 18–21	34.3%	33.5%	81.8%	89.5%	87.0%	2009^32^
56	Plasma	qPCR	EGFR exon 18–21	23.2%	NA	NA	NA	NA	2010^33^
58	Serum	Mutant-enriched PCR	EGFR exon19 + L858R	24.1%	31.0%	77.8%	100.0%	93.1%	2011^34^
86	Serum	ARMS-PCR	EGFR exon 18–21	25.6%	59.3%	43.1%	100.0%	66.3%	2012^35^
264	Plasma	DHPLC	EGFR exon 18–21	34.5%	34.9%* (22/63)	NA	NA	NA	2012^36^
627	Plasma	mutant-enriched liquidchip	EGFR exon 19 + L858R	22.0%	35.6%*(21/59)	NA	NA	NA	2012^37^
111	Plasma	Mutant-enriched PCR and sequencing	EGFR exon19 + L858R	17.1%	40.5%	35.6%	95.5%	71.2%	2013^38^
86	Plasma	ARMS	EGFR exon18–21	31.4%	46.5%	67.5%	100%	84.9%	2013^3^
57	Serum	PNA-LNA PCR clamp	EGFR exon 18–21	19.3%	21.1%	66.7%	93.3%	87.7%	2013^39^
94	Plasma	Scorpion-ARMS	EGFR exon18–21	20.2%	40.4%	50.0%	100.0%	79.8%	2015^40^
121	Plasma	Mutant-enriched PCR and DHPLC	EGFR exon19, 21	34.7%	36.4%	77.3%	89.6%	85.1%	2016^10^
102	Plasma	NGS	70-gene panel	18.6%	23.5%	NA	NA	79.0%	2016^12^
117	Plasma	Digital PCR	EGFR T790M	47.0%**	NA	NA	NA	NA	2016^41^

Only ctDNA detections containing more than 50 samples were summarized in this table. *Detected in non paired tissue samples; **Detected in NSCLC with acquired EGFR-TKI (TKI) resistance.

In summary, actionable genetic alterations were identified in 37.0% of patients with advanced NSCLC by an NGS-based ctDNA assay. Noninvasive NGS-based ctDNA assays might be a potential method for monitoring EGFR-TKI resistance and discovering drug-resistance mechanisms. The NGS-based ctDNA assay might be a comprehensive and efficient strategy that complements tissue analysis and expands the scope of personalized targeted therapies for advanced NSCLC.

## Electronic supplementary material

Supplementary information
