# Publisher Correction: Discovery of targetable genetic alterations in advanced non-small cell lung cancer using a next-generation sequencing-based circulating tumor DNA assay

**DOI:** 10.1038/s41598-018-19648-9

**Published:** 2018-01-15

**Authors:** Helei Hou, Xiaonan Yang, Jinping Zhang, Zhe Zhang, Xiaomei Xu, Xiaoping Zhang, Chuantao Zhang, Dong Liu, Weihua Yan, Na Zhou, Hongmei Zhu, Zhaoyang Qian, Zhuokun Li, Xiaochun Zhang

**Affiliations:** 1 Department of Medical Oncology, The Affiliated Hospital of Qingdao University, Qingdao University, 16 Jiangsu Road, Qingdao, 266005 China; 2 BGI-Qingdao Institute, Qingdao SINO-GERMAN Ecopark, 2877 Tuanjie Road, Qingdao, 266555 China; 3 Department of Experimental Therapeutics, University of Texas, South Campus Research Building 4 (4SCR), Room 4SCR3.2085, 1901 East Road, Houston, Texas 77054 USA; 4 Department of Thoracic Surgery, Qingdao Municipal Hospital, 1 Jiaozhou Road, Qingdao, 266011 China; 5 Department of Medical Oncology, Qingdao Municipal Hospital, 5 Donghai Middle Road, Qingdao, 266071 China; 6 BGI-Shenzhen, Shenzhen, 518083 China; 7 Department of Pathology, The Affiliated Hospital of Qingdao University, Qingdao University, 16 Jiangsu Road, Qingdao, 266005 China; 8 Binhai Genomics Institute, BGI-Tianjin, BGI-Shenzhen, Tianjin, 300308 China

Correction to: *Scientific Reports* 10.1038/s41598-017-14962-0, published online 03 November 2017

The original HTML version of this Article contained a typographical error in the publication date ‘03 November 2017’ which was incorrectly given as ‘06 November 2017’. This has now been corrected in the HTML version of the Article.

