# Supplementary material for: Discovery of targetable genetic alterations in advanced non-small cell lung cancer using a next-generation sequencing-based circulating tumor DNA assay
Source: Sci Rep. 2017 Nov 3;7:14605. doi: 10.1038/s41598-017-14962-0 (PMC5668369; doi:10.1038/s41598-017-14962-0)
Supplement: Supplementary file 1 — Supplementary information [file 41598_2017_14962_MOESM1_ESM.pdf]

Title

Discover targetable genetic alterations in advanced non-small cell lung cancer using next-generation sequencing based circulating tumor DNA array

Authors

Helei Hou[1#], Xiaonan Yang[2#], Jinping Zhang[3], Zhe Zhang[4], Xiaomei Xu[5], Xiaoping Zhang[6], Chuantao Zhang[1], Dong Liu[1], Weihua Yan[7], Na Zhou[1], Hongmei Zhu[8], Zhaoyang Qian[8], Zhuokun Li[2], Xiaochun Zhang [1]*

Affiliations

[1]Department of Medical Oncology, The Affiliated Hospital of Qingdao University, Qingdao University, 16 Jiangsu Road, Qingdao, 266005, China

[2] BGI-Qingdao Institute, Qingdao SINO-GERMAN Ecopark, 2877 Tuanjie Road, Qingdao, 266555, China

[3]Department of Experimental Therapeutics, University of Texas, South Campus Research Building 4 (4SCR), Room 4SCR3.2085, 1901 East Road, Houston, Texas 77054

[4]Department of Thoracic Surgery, Qingdao Municipal Hospital, 1 Jiaozhou Road, Qingdao, 266011, China

[5]Department of Medical Oncology, Qingdao Municipal Hospital, 5 Donghai Middle Road, Qingdao, 266071, China

[6] BGI-Shenzhen, Shenzhen, 518083, China

[7]Department of Pathology, The Affiliated Hospital of Qingdao University, Qingdao University, 16 Jiangsu Road, Qingdao, 266005, China

[8]Binhai Genomics Institute, BGI-Tianjin, BGI-Shenzhen, Tianjin 300308, China

[#] These authors contributed equally to this work.

*Corresponding author: Xiaochun Zhang, Department of Medical Oncology, The Affiliated Hospital of Qingdao University, Qingdao University, 16 Jiangsu Road, Qingdao, 266005, China

Tel:086053282913271

Supplementary Table S1 Gene list for NGS assay in this study

ABL1	C1R	DIS3	FGF19	HSPA4	MIR142	PAX5	RB1	SRSF2
ABL2	C1S	DNMT1	FGF23	IDH1	MITF	PBRM1	REL	SSTR2
ACVR1B	CARD11	DNMT3A	FGF3	IDH2	MLH1	PCBP1	RET	STAG2
ACVR2A	CASP8	DOT1L	FGF4	IFNAR1	MLH3	PCM1	RHEB	STAT4
AJUBA	CBFB	DUSP6	FGF6	IFNAR2	MLL	PDGFRA	RICTOR	STAT5B
AKT1	CBL	EDNRA	FGF7	IGF1	MLL2	PDGFRB	RNASEL	STK11
AKT2	CBLB	EGFR	FGFR1	IGF1R	MLL3	PDK1	RNF43	SUFU
AKT3	CBR1	EGR3	FGFR2	IGF2	MLL4	PHF6	ROBO1	SUZ12
ALK	CCND1	EIF4A2	FGFR3	IKBKB	MPL	PIGF	ROBO2	SYK
ALOX12B	CCND2	ELAC2	FGFR4	IKBKE	MRE11A	PIK3C2A	ROS1	TAF1
ANGPT1	CCND3	ELF3	FH	IKZF1	MS4A1	PIK3C2B	RPA1	TBL1XR1
ANGPT2	CCNE1	EML4	FLCN	IL7R	MSH2	PIK3C2G	RPL22	TBX3
APC	CD79A	EP300	FLT1	INHBA	MSH3	PIK3C3	RPL5	TEK
APCDD1	CD79B	EPCAM	FLT3	IRF4	MSH4	PIK3CA	RPS14	TERT
AR	CDC25C	EPHA2	FLT4	IRS2	MSH5	PIK3CB	RPS6KB1	TET2
ARAF	CDC42	EPHA3	FNTA	ITGB2	MSH6	PIK3CG	RPTOR	TFG
ARFRP1	CDC73	EPHA5	FOXA1	JAK1	MSR1	PIK3R1	RUNX1	TGFBR2
ARHGAP35	CDH1	EPHB1	FOXA2	JAK2	MTOR	PIK3R2	RUNX1T1	TIPARP
ARID1A	CDK12	EPHB2	FOXL2	JAK3	MUC1	PLK1	RXRA	TLR4
ARID1B	CDK2	EPHB6	FPGS	JUN	MUTYH	PML	RXRB	TMEM127
ARID2	CDK4	EPPK1	FUBP1	KAT6A	MYC	PMS1	RXRG	TNFAIP3
ARID5B	CDK6	ERBB2	FYN	KDM5A	MYCL1	PMS2	SDHAF2	TNFRSF14
ASXL1	CDK8	ERBB3	GAB2	KDM5C	MYCN	PNRC1	SDHB	TNFRSF8
ATM	CDKN1A	ERBB4	GATA1	KDM6A	MYD88	POLQ	SDHC	TNFSF11
ATR	CDKN1B	ERCC2	GATA2	KDR	NAV3	PPP2R1A	SDHD	TNFSF13B
ATRX	CDKN2A	ERCC3	GATA3	KEAP1	NBN	PRDM1	SEMA3A	TOP1
AURKA	CDKN2B	ERG	GID4	KIF1B	NCOA1	PRKAA1	SEMA3E	TOP2A
AURKB	CDKN2C	ESR1	GNA11	KIF5B	NCOA2	PRKAR1A	SETBP1	TOP2B
AXIN1	CDX2	ETV1	GNA13	KIT	NCOR1	PRKCA	SETD2	TP53
AXIN2	CEBPA	ETV6	GNAQ	KLF4	NEK11	PRKCB	SF1	TRAF7
AXL	CFLAR	EWSR1	GNAS	KLHL6	NF1	PRKCG	SF3B1	TSC1
B2M	CHD1	EXT1	GNRHR	KRAS	NF2	PRKDC	SH2B3	TSC2
B4GALT3	CHD2	EXT2	GPR124	LCK	NFE2L2	PRSS8	SIN3A	TSHR
BACH1	CHD4	EZH2	GRIN2A	LIMK1	NFE2L3	PSMB1	SLAMF7	TSHZ2
BAK1	CHEK1	FAM123B	GRM3	LRRK2	NFKBIA	PSMB2	SLC4A1	TSHZ3
BAP1	CHEK2	FAM46C	GSK3B	LYN	NKX2-1	PSMB5	SLIT2	TUBA1A
BARD1	CHUK	FANCA	H3F3A	MALAT1	NKX3-1	PTCH1	SMAD2	TUBB
BCL2	CIC	FANCC	H3F3C	MAP2K1	NOTCH1	PTCH2	SMAD3	TUBD1
BCL2A1	CRBN	FANCD2	HCK	MAP2K2	NOTCH2	PTEN	SMAD4	TUBE1
BCL2L1	CREBBP	FANCE	HDAC1	MAP2K4	NOTCH3	PTP4A3	SMARCA1	TUBG1

BCL2L11	CRIPAK	FANCF	HDAC2	MAP3K1	NOTCH4	PTPN11	SMARCA4	TYR
BCL2L2	CRKL	FANCG	HDAC3	MAP3K13	NPM1	PTPRD	SMARCB1	U2AF1
BCL6	CRLF2	FANCI	HDAC4	MAPK1	NR3C1	RAC1	SMARCD1	USP9X
BCOR	CROT	FANCL	HDAC6	MAPK3	NRAS	RAC2	SMC1A	VEGFA
BCORL1	CSF1R	FANCM	HDAC8	MAPK8	NSD1	RAD21	SMC3	VEGFB
BCR	CTCF	FAT3	HGF	MAPK8IP1	NTRK1	RAD50	SMO	VEZF1
BLM	CTLA4	FBXW7	HIF1A	MAX	NTRK2	RAD51	SOCS1	VHL
BMPR1A	CTNNA1	FCGR1A	HIST1H1C	MC1R	NTRK3	RAD51B	SOX10	WHSC1L1
BRAF	CTNNB1	FCGR2A	HIST1H2BD	MCL1	NUP93	RAD51C	SOX17	WISP3
BRCA1	CUL4A	FCGR2B	HIST1H3B	MDM2	PAK3	RAD51D	SOX2	WWP1
BRCA2	CUL4B	FCGR2C	HNF1A	MDM4	PAK7	RAD52	SOX9	XIAP
BRIP1	CYLD	FCGR3A	HRAS	MECOM	PALB2	RAD54L	SPEN	XPA
BTG1	CYP17A1	FCGR3B	HRH2	MED12	PARP1	RAF1	SPOP	XPC
BTK	DAXX	FGF10	HSD17B3	MEF2B	PARP2	RARA	SPRY4	XPO1
C11orf30	DDR1	FGF12	HSD3B2	MEN1	PARP3	RARB	SRC	XRCC3
C1QA	DDR2	FGF14	HSP90AA1	MET	PARP4	RARG	SRD5A2	YES1
ZNF217	ZNF703	ZRSR2	WT1					

Supplementary Table S2 The basic summary of the sequencing data via NGS based ctDNA assay

	**Range**	**mean±SEM**
Depth	600.57-1636.07	944.69±337.01
Mapping rate	96.74%-99.60%	98.04%±0.82%
On target rate	25.48%-74.63%	51.93%±12.74%
1×coverage	99.62%-99.99%	99.85%±0.12%
100×coverage	96.07%-99.34%	97.95%±0.89%

Supplementary Table S3. Targetable genomic alterations identified in 44 NSCLC patients by NGS based ctDNA assay

Samples	Age	Stage	Sex	Pathology	Gene	cHGVs	pHGVs	Frequency
1	46	IV	F	AC	EGFR	c.2573T>G	p.L858R	1.40%
1	52	IV	M	AC	EGFR	c.2573T>G	p.L858R	7.10%
3	39	IV	M	AC	RET	KIF5B-RET	KIF5B-RET	1.80%
4	47	IV	M	AC	BRAF	c.1799T>A	p.V600E	22.50%
					EGFR	c.2237_2257delAATTAAGAGAAGCAACATCTCinsTGT	p.E746_P753delinsVS	1.30%
5	69	IV	F	AC	EGFR	c.2235_2249delGGAATTAAGAGAAGC	p.E746_A750del	50.90%
					NF1	c.3088_3089delTC	p.S1030Ifs*8	11.50%
					TP53	c.238_248delCCTACACCGGC	p.P80Gfs*65	24.50%
6	78	IIIB	F	AC	BRAF	c.1799T>A	p.V600E	1.20%
7	65	IV	F	AC	EGFR	c.2573T>G	p.L858R	56.90%
8	55	IIIB	F	AC	KRAS	c.34G>T	p.G12C	1.40%
9	42	IV	F	AC	STK11	c.178_179insT	p.Y60Lfs*103	9.70%
10	48	IV	F	AC	PTEN	c.388C>T	p.R130*	0.50%
11	68	IV	F	AC	EGFR	c.2235_2249delGGAATTAAGAGAAGC	p.E746_A750del	0.50%
12	76	IV	M	AC	BRAF	c.1406G>C	p.G469A	7.20%
					TP53	c.423C>A	p.C141*	8.50%
13	83	IV	M	AC	KRAS	c.35G>A	p.G12D	6.90%
14	76	IV	M	SCC	ATM	c.7345_7348delGAAT	p.E2449*	1.10%
15	55	IV	M	AC	EGFR	c.2235_2249delGGAATTAAGAGAAGC	p.E746_A750del	8.00%
16	42	IV	M	SCC	EGFR	c.2573T>G	p.L858R	0.70%
					PIK3CA	c.3140A>G	p.H1047R	1.60%
17	46	IV	F	*SCC*	FBXW7	c.1514G>A	p.R505H	0.60%
					FBXW7	c.1394G>A	p.R465H	5.50%
18	58	IV	M	AC	EGFR	c.2573T>G	p.L858R	11.00%
					NRAS	c.181C>A	p.Q61K	0.60%
19	38	IV	M	AC	EGFR	c.2236_2250delGAATTAAGAGAAGCA	p.E746_A750del	56.50%
20	46	IIIB	M	AC	KRAS	c.34G>T	p.G12C	0.50%
21	51	IV	F	AC	EGFR	c.2236_2250delGAATTAAGAGAAGCA	p.E746_A750del	0.60%
22	65	IV	F	AC	EGFR	c.2235_2249delGGAATTAAGAGAAGC	p.E746_A750del	1.30%
23	43	IV	F	AC	FBXW7	c.1514G>A	p.R505H	0.50%
24	56	IIIB	F	AC	FBXW7	c.1514G>A	p.R505H	0.40%
					PIK3CA	c.3129G>A	p.M1043I	17.50%
25	63	IV	M	AC	EGFR	c.2235_2249delGGAATTAAGAG	p.E746_A750del	0.60%

						AAGC		
					FBXW7	c.1513C>A	p.R505S	0.50%
26	71	IV	M	AC	BRAF	c.1742A>G	p.N581S	11.20%
27	33	IV	F	AC	ALK	EML4-ALK	-	1.27%
28	65	IV	F	AC	EGFR	c.2156G>C	p.G719A	0.80%
29	57	IV	F	AC	NF2	c.331C>T	p.Q111*	15.90%
					TP53	c.548C>G	p.S183*	17.70%
30	46	IV	F	AC	EGFR	c.2300_2308dupCCAGCGTGG	p.A767_V769dup	28.20%
					FBXW7	c.1514G>A	p.R505H	0.50%
31	54	IV	F	AC	EGFR	c.2573T>G	p.L858R	12.10%
					TP53	c.1024C>T	p.R342*	1.20%
32	66	IV	M	AC	KRAS	c.35G>A	p.G12D	1.20%
33	63	IV	F	AC	EGFR	c.2217_2234dupAATTCCCGTCG CTATCAA	p.I740_K745dup	14.40%
34	77	IV	M	AC	EGFR	c.2573T>G	p.L858R	2.70%
35	70	IIIB	M	SCC	TP53	c.716_719delACAG	p.N239Ifs*7	12.50%
36	58	IV	F	SCC	EGFR	c.2573T>G	p.L858R	1.80%
37	69	IV	M	AC	MET	c.3742T>C	p.Y1248H	13.80%
38	56	IV	M	AC	EGFR	c.2497T>G	p.L833V	3.00%
					EGFR	c.2573T>G	p.L858R	3.20%
39	48	IV	M	AC	BRAF	c.1406G>T	p.G469V	0.80%
40	55	IV	M	AC	EGFR	c.2303G>T	p.S768I	0.60%
41	57	IV	M	AC	EGFR	c.2504A>T	p.H835L	5.80%
42	58	IV	F	AC	EGFR	c.2303G>T	p.S768I	6.90%
					EGFR	c.2156G>C	p.G719A	0.80%
43	57	IV	M	AC	EGFR	c.2235_2252delGGAATTAAGAG AAGCAACinsAAT	p.E746_T751delinsI	0.50%
44	61	IV	F	AC	EGFR	c.2235_2249delGGAATTAAGAG AAGC	p.E746_A750del	7.70%

Abbreviations: AC , adenocarcinoma; SCC, squamous cell cancer
